# Supplementary material for: Stabilization of supramolecular membrane protein–lipid bilayer assemblies through immobilization in a crystalline exoskeleton
Source: Nat Commun. 2021 Apr 13;12:2202. doi: 10.1038/s41467-021-22285-y (PMC8044103; doi:10.1038/s41467-021-22285-y)
Supplement: Supplementary file 1 — Supplementary Information [file 41467_2021_22285_MOESM1_ESM.docx]

*Supplementary Information*

**Stabilization of Supramolecular Membrane Protein-Lipid Bilayer Assemblies Through Immobilization in a Crystalline Exoskeleton**

Fabian C. Herbert,^1^ Sameera S. Abeyrathna,^1^ Nisansala S. Abeyrathna,^1^ Yalini H. Wijesundara,^1^ Olivia R. Brohlin,^1^ Francesco Carraro, ^2^ Heinz Amenitsch, ^3^ Paolo Falcaro, ^2^ Michael A. Luzuriaga,^1^ Alejandra Durand-Silva,^1^ Shashini D. Diwakara,^1^ Ronald A. Smaldone,^1^ Gabriele Meloni,^1 *^and Jeremiah J. Gassensmith,^1,4, *^

^1^Department of Chemistry and Biochemistry, The University of Texas at Dallas, 800 West Campbell Road, Richardson, TX 75080. ^2^Institute of Physical and Theoretical Chemistry, Graz University of Technology, Stremayrgasse 9, Graz 8010, Austria. ^3^Institute of Inorganic Chemistry, Graz University of Technology, Stremayrgasse 9, Graz 8010, Austria. ^4^ Department of Bioengineering, The University of Texas at Dallas, 800 West Campbell Road, Richardson, TX 75080, USA.

# Supplementary Figures and Tables

##

## Supplementary Table 1. BET surface area of Lp@ZIF bio-composites

| Sample ID | BET surface (m^2^/g) |
| --- | --- |
| 20*×*16 ZIF-L | *73* |
| 20*×*16 Lp@ZIF | 38 |
| 40*×*16 ZIF-L | 385 |
| 40*×*16 Lp@ZIF | 288 |
| 40*×*16 IroT@ZIF | 26 |

## Supplementary Table 2. Blank liposome dynamic-light scattering (DLS) analysis.

| Sample ID | Size (nm) | PDI |
| --- | --- | --- |
| Lp (p) | 141.2 | 0.138 |
| Lp *(25 ^ͦ^ C)* | 291.7 | 0.318 |
| Lp *(55 ^ͦ^ C)* | 604.8 | 0.449 |
| Lp *(80 ^ͦ^ C)* | 675.6 | 0.465 |
| 20*×*16 Lp@ZIF (25 ^ͦ^ C) | 120.6 | 0.147 |
| 20*×*16 Lp@ZIF *(55 ^ͦ^ C)* | 125.8 | 0.211 |
| 20*×*16 Lp@ZIF *(80 ^ͦ^ C)* | 122.5 | 0.181 |
| 40*×*16 Lp@ZIF *(25 ^ͦ^ C)* | 124.9 | 0.109 |
| 40*×*16 Lp@ZIF *(55 ^ͦ^ C)* | 121.2 | 0.187 |
| 40*×*16 Lp@ZIF *(80 ^ͦ^ C)* | 119.3 | 0.232 |

## Supplementary Table 3. CopA proteoliposome dynamic-light scattering (DLS) analysis.

| Sample ID | Size (nm) | PDI |
| --- | --- | --- |
| CopA-PL (p) | 160.6 | 0.166 |
| CopA-PL *(RT)* | 360.8 | 0.306 |
| CopA-PL *(55 ^ͦ^ C)* | 325.9 | 0.542 |
| CopA-PL *(80 ^ͦ^ C)* | 326.1 | 0.429 |
| 20*×*16 CopA-PL@ZIF *(25 ^ͦ^ C)* | 126.8 | 0.152 |
| 20*×*16 CopA-PL@ZIF *(55 ^ͦ^ C)* | 128.9 | 0.138 |
| 20*×*16 CopA-PL@ZIF *(80 ^ͦ^ C)* | 125.8 | 0.122 |
| 40*×*16 CopA-PL@ZIF *(25 ^ͦ^ C)* | 127.8 | 0.145 |
| 40*×*16 CopA-PL@ZIF *(55 ^ͦ^ C)* | 139.4 | 0.189 |
| 40*×*16 CopA-PL@ZIF *(80 ^ͦ^ C)* | 318.8 | 0.475 |

## Supplementary Table 4. IroT proteoliposome dynamic-light scattering (DLS) analysis

| Sample ID | Size (nm) | PDI |
| --- | --- | --- |
| IroTPL (p) | 218.1 | 0.113 |
| IroTPL (*RT)* | 291.7 | 0.318 |
| IroTPL *(55 ^ͦ^ C)* | 632.9 | 0.536 |
| IroTPL *(80 ^ͦ^ C)* | 528.4 | 0.571 |
| 40*×*16 IroTPL@ZIF *(25 ^ͦ^ C)* | 199.2 | 0.165 |
| 40*×*16 IroTPL@ZIF *(55 ^ͦ^ C)* | 196.3 | 0.145 |
| 40*×*16 IroTPL@ZIF *(80 ^ͦ^ C)* | 195.6 | 0.14 |


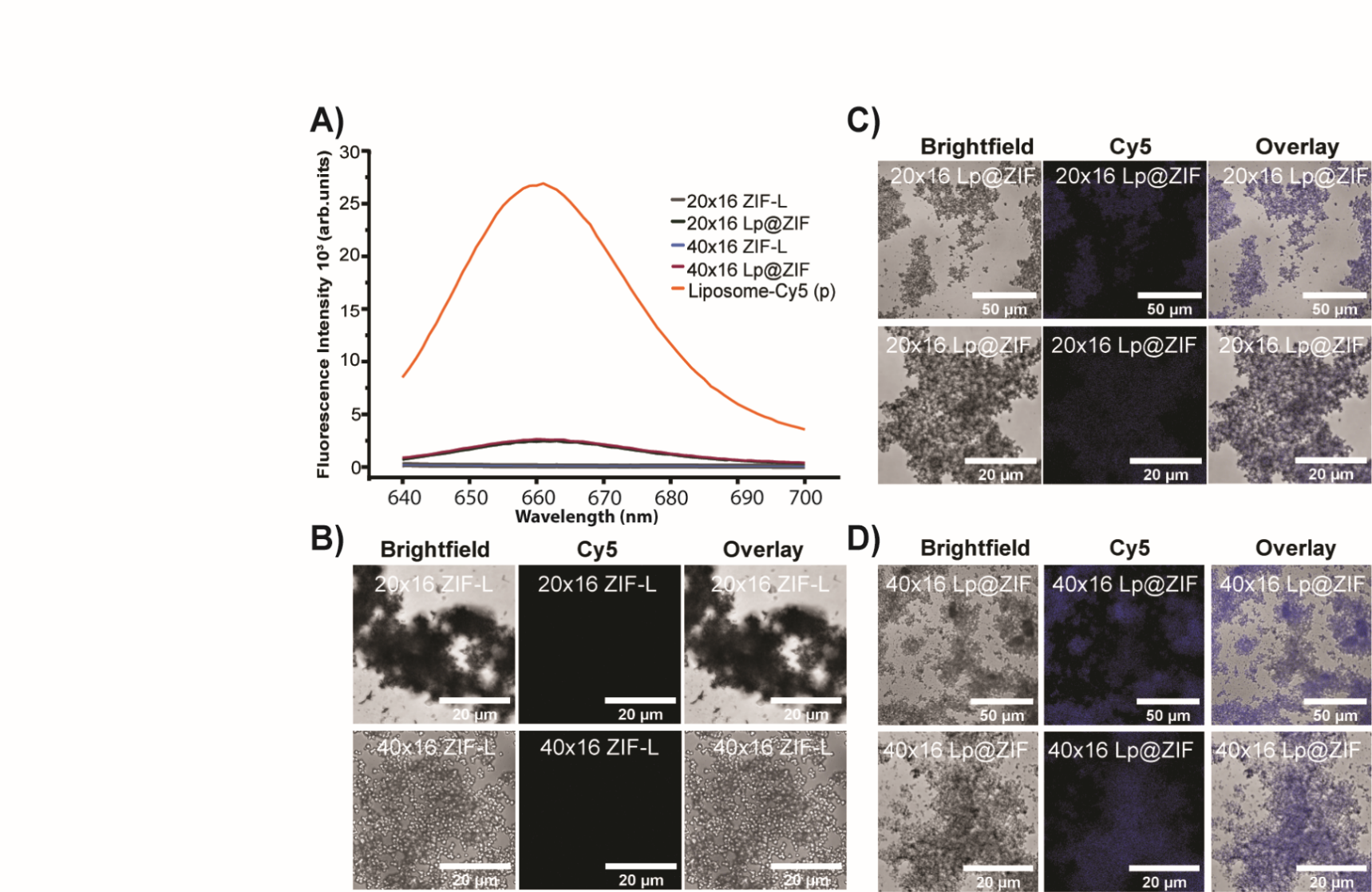


**Supplementary** Figure 1. Encapsulation efficiency was determined by fluorescence on the residual supernatant upon liposome immobilization in ZIF. A) Fluorescence emission spectra of Cy5-loaded liposome starting solution (orange line) versus supernatants collected after the 20*×*16 Lp@ZIF (green), 40*×*16 Lp@ZIF (magenta) syntheses and removal by centrifugation. Controls include supernatants collected for both 20*×*16 (grey) and 40*×*16 ZIF-L (blue). Y-axis expressed as arbitrary units (arb.units). B) Confocal microscope images of 20*×*16 and 40*×*16 ZIF-L, C) 20*×*16 Lp@ZIF, and D) 40*×*16 Lp@ZIF. Pristine ZIF-L shows no intrinsic fluorescence when imaged under the Cy5 channel. On the other hand, both 20*×*16 Lp@ZIF and 40*×*16 Lp@ZIF prepared with Cy5-loaded liposomes are fluorescent (670 nm).


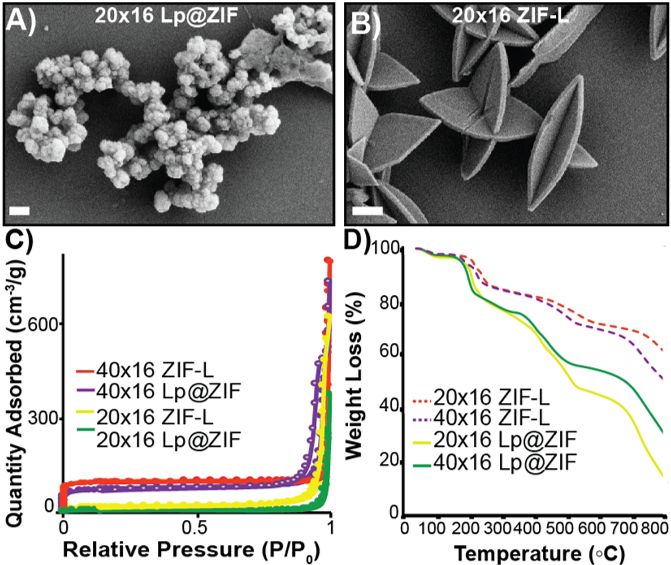


**Supplementary** Figure 2. Characterization of ZIF-L and ZIF-L liposome composites. SEM micrograph of A) 20*×*16 Lp@ZIF (Scale bar = 200 nm) and B) pristine 20*×*16 ZIF (Scale bar = 1 µm). C) Nitrogen isotherms of 40*×*16 Lp@ZIF (purple), 40*×*16 ZIF-L (orange), 20*×*16 ZIF-L (yellow), and 20*×*16 Lp@ZIF (green). D) Thermogravimetric analysis of 40*×*16 Lp@ZIF (green), 40*×*16 ZIF-L (purple-dashed line), 20*×*16 ZIF-L (orange-dashed line), and 20*×*16 Lp@ZIF (yellow).


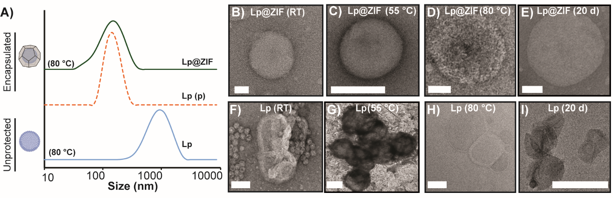


**Supplementary** Figure 3. Characterization of 40*×*16 Lp@ZIF. A) DLS of exfoliated 40*×*16 Lp@ZIF liposomes, pristine, and stressed non-encapsulated liposomes. Color scheme: 40*×*16 Lp@ZIF 80 ^ͦ^ C (green), Lp pristine (orange-dashed line), and unencapsulated control at 80 ^ͦ^ C (light-blue). TEM of micrographs of B) exfoliated liposome after exposure to RT for 48 h (Scale bar = 50 nm), C) exfoliated liposome after exposure to 55 ^ͦ^ C for 15 min (Scale bar = 100 nm), D) exfoliated liposome after exposure to 80 ^ͦ^ C for 5 min (Scale bar = 50 nm), and E) exfoliated liposome after incubation at RT for 20 days (Scale bar = 50 nm). TEM micrographs of F) non-encapsulated liposome after exposure to 25 ^ͦ^ C for 48 h (Scale bar = 200 nm), G) non-encapsulated liposome after exposure to 55 ^ͦ^ C for 15 min (Scale bar = 200 nm), H) non-encapsulated liposome after exposure to 80 ^ͦ^ C for 5 min(Scale bar = 200 nm), and I) non-encapsulated liposome after incubation at RT for 20 days (Scale bar = 500 nm).


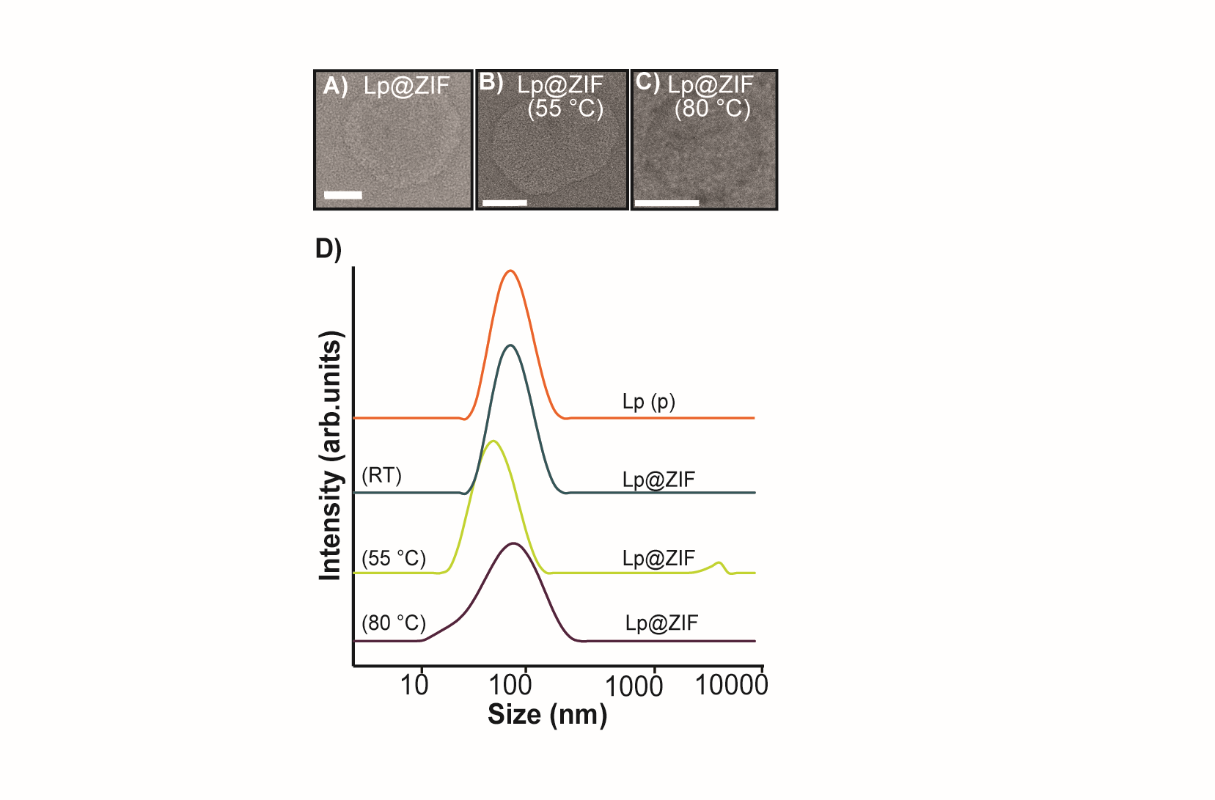


**Supplementary** Figure 4. Characterization of 20*×*16 Lp@ZIF. A) TEM micrographs of exfoliated 20*×*16 Lp@ZIF stressed at room temperature (Scale bar = 50 nm), B) 55 ^ͦ^ C for 15 min (Scale bar = 100 nm), C) and 80 ^ͦ^ C for 5 min (Scale bar = 50 nm). Scale bars = 100 nm. D) DLS of exfoliated 20*×*16 Lp@ZIF, stressed non-encapsulated liposomes, and pristine liposomes. Color scheme: Lp pristine (orange), 20*×*16 Lp@ZIF RT (blue), 20*×*16 Lp@ZIF 55 ^ͦ^ C (green), 20*×*16 Lp@ZIF 80 ^ͦ^ C (purple). Y-axis expressed as arbitrary units (arb.units).


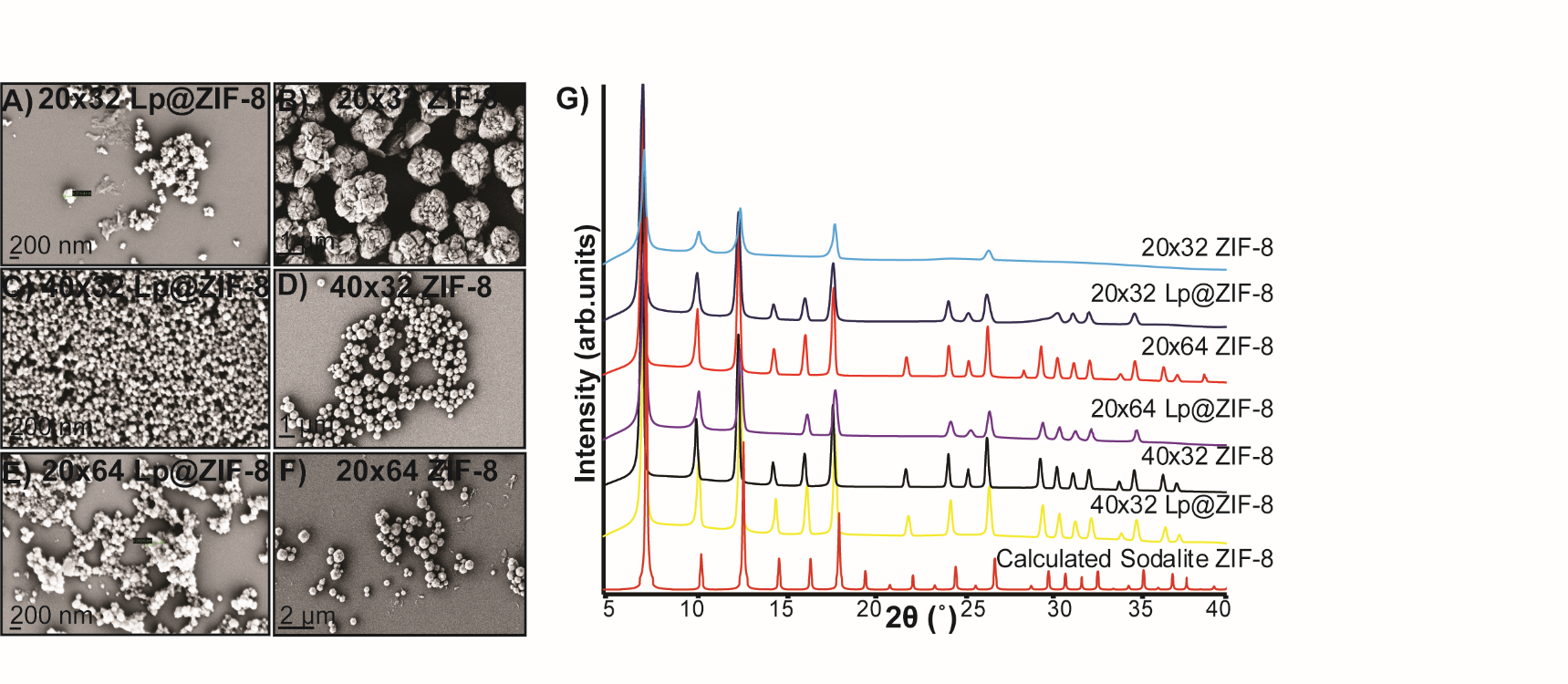


**Supplementary** Figure 5: Crystal characterization of 20*×*32, 40*×*32, and 20*×*64 Lp@ZIF-8. A) SEM micrograph of 20*×*32 Lp@ZIF-8 and B) of 20*×*32 pristine ZIF-8.C) SEM micrograph of 40*×*32 Lp@ZIF-8 and D) of 40*×*32 pristine ZIF-8. E). SEM micrograph of 20*×*64 Lp@ZIF-8 and F) of 20*×*64 pristine ZIF-8. G) PXRD spectra collected from 20*×*32 Lp@ZIF-8, 40*×*32 Lp@ZIF-8, 20*×*64 Lp@ZIF-8, and corresponding controls. Color scheme: 20*×*32 ZIF-8 (light-blue), 20*×*32 Lp@ZIF-8 (dark-blue), 20*×*64 ZIF-8 (orange), 20*×*64 Lp@ZIF-8 (purple), 40*×*32 ZIF-8 (black), 40*×*32 Lp@ZIF-8 (yellow), calculated sodalite ZIF-8 (red).Y-axis expressed as arbitrary units (arb.units).


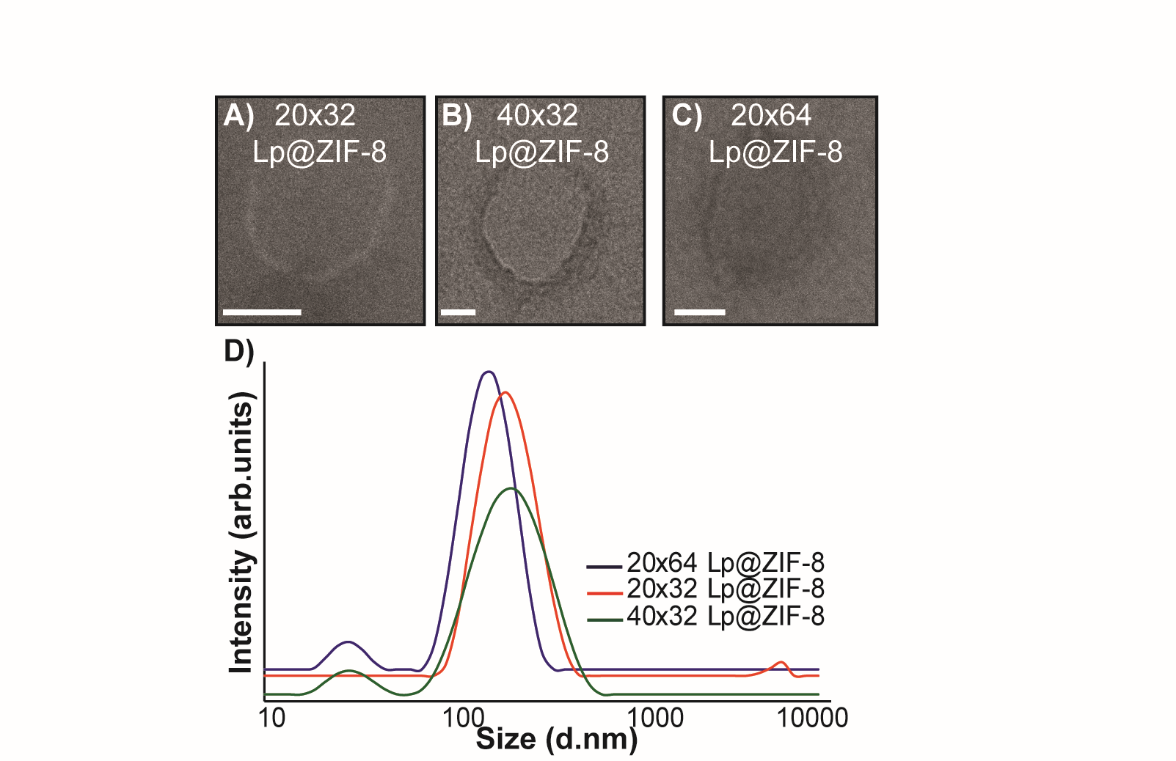


**Supplementary** Figure 6: Liposome recovery of 20*×*32, 40*×*32, and 20*×*64 Lp@ZIF-8. A) TEM micrograph of liposomes recovered after exfoliation of 20*×*32 Lp@ZIF-8 (Scale bar = 100 nm), B) of liposomes recovered after exfoliation of 40*×*32 Lp@ZIF-8 (Scale bar = 50 nm), and C) of liposomes recovered after exfoliation of 20*×*64 Lp@ZIF-8 (Scale bar = 100 nm). E). DLS exfoliated 20*×*32 Lp@ZIF-8 (orange), 40*×*32 Lp@ZIF-8 (green), and 20*×*64 Lp@ZIF-8 (purple). . Y-axis expressed as arbitrary units (arb.units).


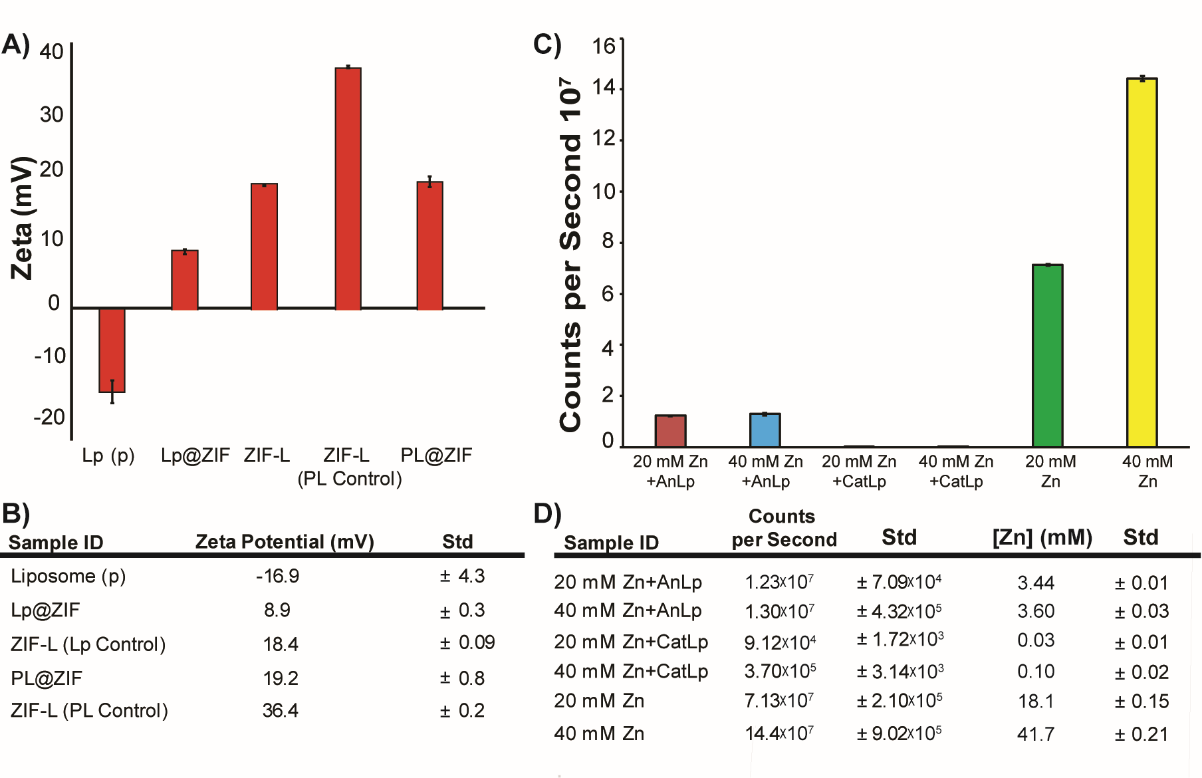


**Supplementary** Figure 7: Investigation of ZIF growth around liposome and proteoliposome formulations. A) Zeta potential plot of pristine liposomes, encapsulated liposomes, and ZIF-L control. Data also includes measurement of proteoliposomes@ZIF and the respective control. Y-axis units expressed as millivolt. B) Zeta potential values of pristine liposomes, encapsulated liposomes, ZIF-L control, proteoliposomes@ZIF, ZIF-L (Proteoliposome control). Error =standard deviation(n=3). C) ICPMS Zn count plot for anionic (AnLp) and cationic (CatLp) incubated in varying concentrations of the metal precursor. Color scheme: 20 mM Zn+ AnLp (red), 40 mM Zn+ AnLp (blue), 20 mM Zn (green), and 40 mM Zn (yellow). Error bars =standard deviation(n=3). D) Count per second and Zn concentrations (mM) for anionic (AnLp) and cationic (CatLp) liposomes exposed to Zn solutions. Error =standard deviation(n=3).


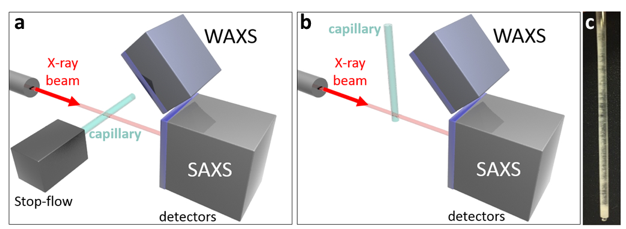


**Supplementary** Figure 8: SAXs experimental Setup. Schematic view of the capillary (sample), X-ray beam and detectors spatial arrangement in the (a) stop-flow set-up and (b) in the “vertically positioned capillary” set-up and a picture of the 20*×*16 Lp@ZIF sample precipitated at the bottom of the (c) vertically mounted capillary picture taken 30 min after mixing the precursors).


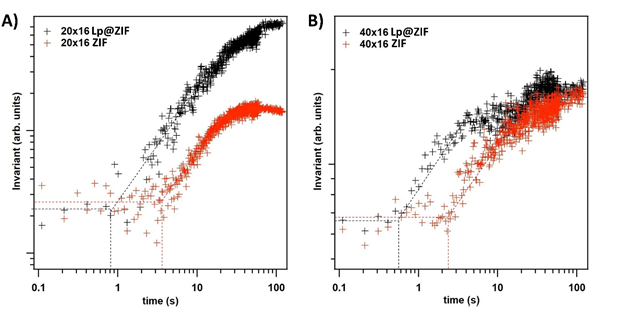


**Supplementary** Figure 9: Time evolution of Porod-Invariant Q̃ (0.1–0.6 nm^−1^ range) calculated from time‐resolved SAXS synthesis of (a) 20×16 Lp@ZIF (black) and 20×16 ZIF (orange) and (b) 40×16 Lp@ZIF (black) and 40×16 ZIF (orange). Selected SAXS patterns used for the calculation of Q̃ are reported in Figure S17. The dashed lines are plotted to highlight the starting time of the Q̃ increase.


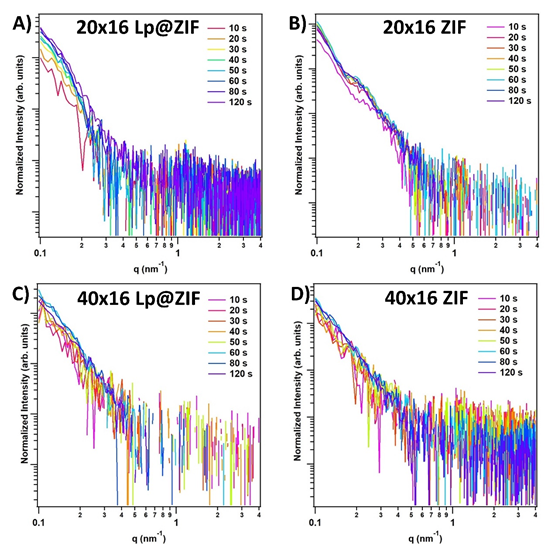


**Supplementary** Figure 10: Time evolution of SAXS patterns (background subtracted) from time‐resolved SAXS synthesis of A) 20×16 Lp@ZIF. 10 s (magenta), 20 s (dark-yellow), 30 s (light-yellow), 40 s (green), 50 s (blue), 60 s (dark-blue), 80 s (light-purple), 120 s (dark-purple). B) 20×16 ZIF. 10 s (magenta), 20 s (dark-purple), 30 s (dark-orange), 40 s (light-orange), 50 s (light-green), 60 s (blue), 80 s (dark-blue), 120 s (purple). C) 40×16 Lp@ZIF. 10 s (magenta), 20 s (pink), 30 s (dark-orange), 40 s (light-orange), 50 s (light-green), 60 s (blue), 80 s (dark-blue), 120 s (purple). D) 40×16 ZIF. 10 s (magenta), 20 s (dark-purple), 30 s (dark-orange), 40 s (light-orange), 50 s (light-green), 60 s (blue), 80 s (dark-blue), 120 s (purple).

**Supplementary** Table 5: Summary of the particle growth kinetics obtained from the analysis of the time evolution of Porod-Invariant Q̃ (0.1–0.6 nm^−1^ range, Figure S16). Time zero is referred to the end of the precursors mixing.

| **Sample** | **Particle growth start after (s)** | **Particle growth approach plateau after (s)** |
| --- | --- | --- |
| 20*×*16 Lp@ZIF | 0.8 | 25 |
| 20*×*16 ZIF-L | 4 | 40 |
| 40*×*16 Lp@ZIF | 0.6 | 5 |
| 40*×*16 ZIF-L | 2.6 | 25 |


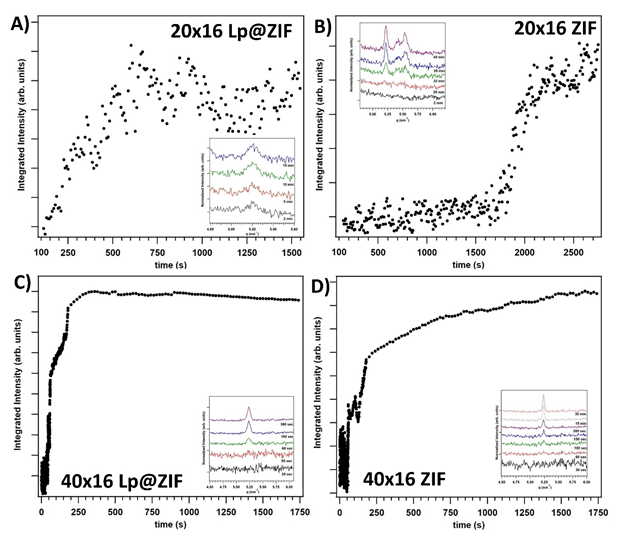


**Supplementary** Figure 11: Time evolution of the integrated intensity of (200) diffraction peak of ZIF-L (5.25 nm^−1^) calculated from time‐resolved SAXS synthesis of 20×16 Lp@ZIF (a), 20×16 ZIF (b), 40×16 Lp@ZIF (c) and 40×16 ZIF (d). In the insets, selected diffraction patterns highlighting the time-evolution of the (200) diffraction peak of ZIF-L (5.25 nm^−1^) are reported. Color scheme of panel A: 2 min (black), 5 min (orange), 10 min (green), and 15 min (blue), of B: 2 min (black), 29 min (orange), 32 min (green), and 35 min (blue), and 40 min (purple). Time zero is referred to the end of the precursors mixing.

**Supplementary** Table 6: Summary of the particle crystallization kinetics obtained from the analysis of the time evolution of the integrated intensity of (200) diffraction peak of ZIF-L (5.25 nm^−1^); Figure S17). Time zero is referred to the end of the precursors mixing.

| **Sample** | **First detection of ZIF 1^st^ diffraction peak (time after precursors mixing)** | **End of crystallization process (plateau of the integrated intensity of ZIF 1^st^ diffraction peak; time after precursors mixing)** |
| --- | --- | --- |
| 20*×*16 Lp@ZIF | <120 seconds | 10 minutes |
| 20*×*16 ZIF-L | 29 minutes | 35 minutes |
| 40*×*16 Lp@ZIF | 50 seconds | 4 minutes |
| 40*×*16 ZIF-L | 60 seconds | 12 minutes |


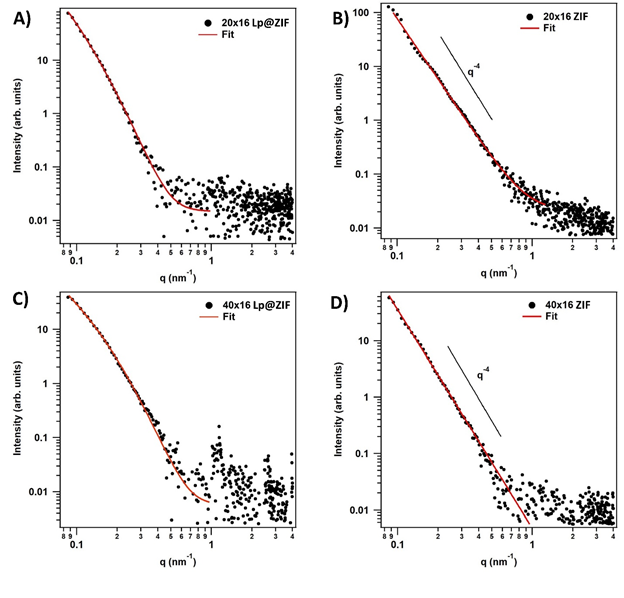


**Supplementary** Figure 12: SAXS patterns (background subtracted) and fitted data 60 s after mixing the precursors of (a) 20×16 Lp@ZIF, (b) 20×16 ZIF, (c) 40×16 Lp@ZIF and (d) 40×16 ZIF. In c and d, the theoretical Porod power law (y=q^-4^) is plotted for comparison.


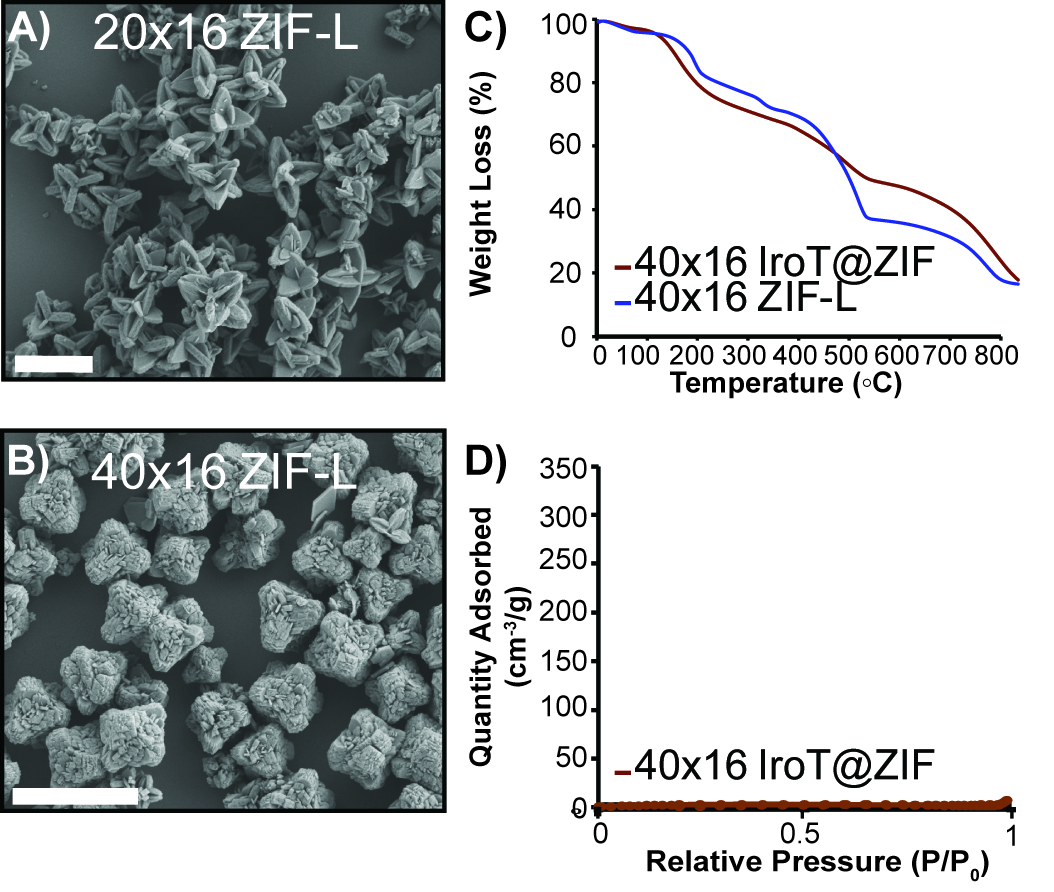


**Supplementary** Figure 13. Characterization of pristine ZIF-L and IroT@ZIF. SEM micrograph of A) 20*×*16 ZIF-L and B) of pristine 40*×*16 ZIF-L (Scale bar = 1 µm). C) TGA of 40*×*16 IroT@ZIF-L (brown) and 40*×*16 ZIF-L control (blue). D) Nitrogen isotherms of 40*×*16 IroT@ZIF-L (brown).


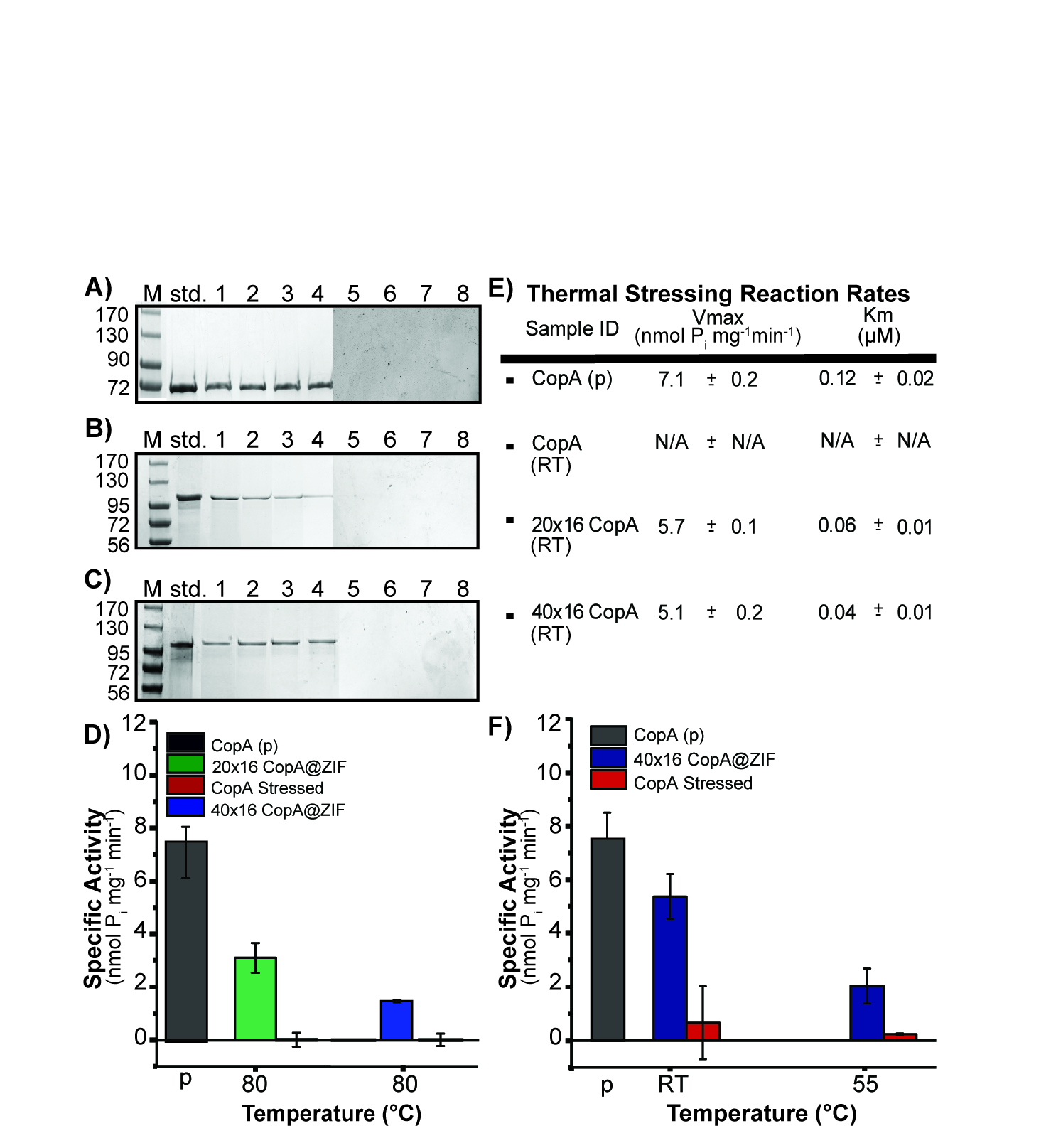


**Supplementary** Figure 14. Characterization of IroT/CopA@ZIF. A–C) Encapsulation efficiency quantification via gel densitometry analysis. Lanes 1-4 and the corresponding lanes 5-8 have been assembled form two separate gel images: A Exfoliated 40*×*16 IroT@ZIF (1)40×16 IroT@ZIF at RT 48 h,(2) 40×16 IroT@ZIF stressed at 55 °C, (3)40×16 IroT@ZIF stressed at 80 °C (4)Non-encapsulated IroT at RT 48 h, (5) 40×16 IroT@ZIF dried supernatant, (6 ) 40×16 IroT@ZIF at RT 48 h supernatant , (7) 40×16 IroT@ZIF stressed at 55 °C supernatant, (8) 40×16 IroT@ZIF stressed at 80 °C supernatant B) 20*×*16 CopA@ZIF,(1) 20×16 CopA@ZIF stressed at RT(2) 20×16 CopA@ZIF stressed at 55 °C (3)20×16 CopA@ZIF stressed at 80 °C (4) 20×16 CopA@ZIF dried (5)-(8) supernatants collected for the 20×16 CopA@ZIF composites shown in lanes 1-4 C) 40*×*16 CopA@ZIF (1) 40×16 CopA@ZIF stressed at RT(2) 40×16 CopA@ZIF stressed at 55 °C (3)40×16 CopA@ZIF stressed at 80 °C (4) 40×16 CopA@ZIF dried (5)-(8) supernatants collected for the 40×16 CopA@ZIF composites shown in lanes 1-4 Standard includes a 0.500 mg mL^-1^ of either IroT or CopA pristine solutions. Full gel images are reported in Supplementary Figure 20 D) Specific activity of exfoliated 20*×*16 (green) and 40*×*16 CopA@ZIF (blue) stressed at 80 °C. Error bars=standard deviation (n=3). Controls include pristine CopA (grey) and non-encapsulated CopA (red) stressed at 80 °C. E) Thermal stressing reaction rates. F) CopA activity of 40*×*16 bio-composites (blue) stressed at 25 °C and 55 °C, respectively. Controls include non-encapsulated stressed CopA (grey) and pristine CopA (red). Error bars=standard deviation (n=3).


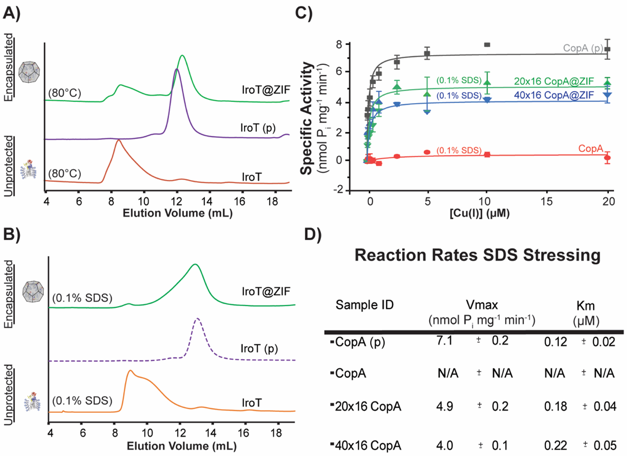


**Supplementary** Figure 15. Characterization of IroT/CopA-detergent complexes ZIF bio-composites. FPLC characterization of A) exfoliated 40*×*16 IroT@ZIF stressed at 80 °C. Color scheme: 40*×*16 IroT@ZIF stressed at 80 °C (green), IroT pristine (purple), and nonencapsulated IroT at 80 °C (orange). B) Exfoliated 40*×*16 IroT@ZIF stressed with 0.1% SDS (green). Controls include non-encapsulated stressed IroT (orange) and pristine IroT (purple-dashed line). Reported absorbances were measured at 280 nm. C) Specific activity of SDS stressed CopA@ZIF bio-composites. Error bars=standard deviation (n=2). Color scheme: CopA pristine (Gray), 40*×*16 CopA@ZIF (blue), 20*×*16 CopA@ZIF (green), with unencapsulated control (red line). D) Reaction rates obtained for SDS stressed bio-composites and controls.


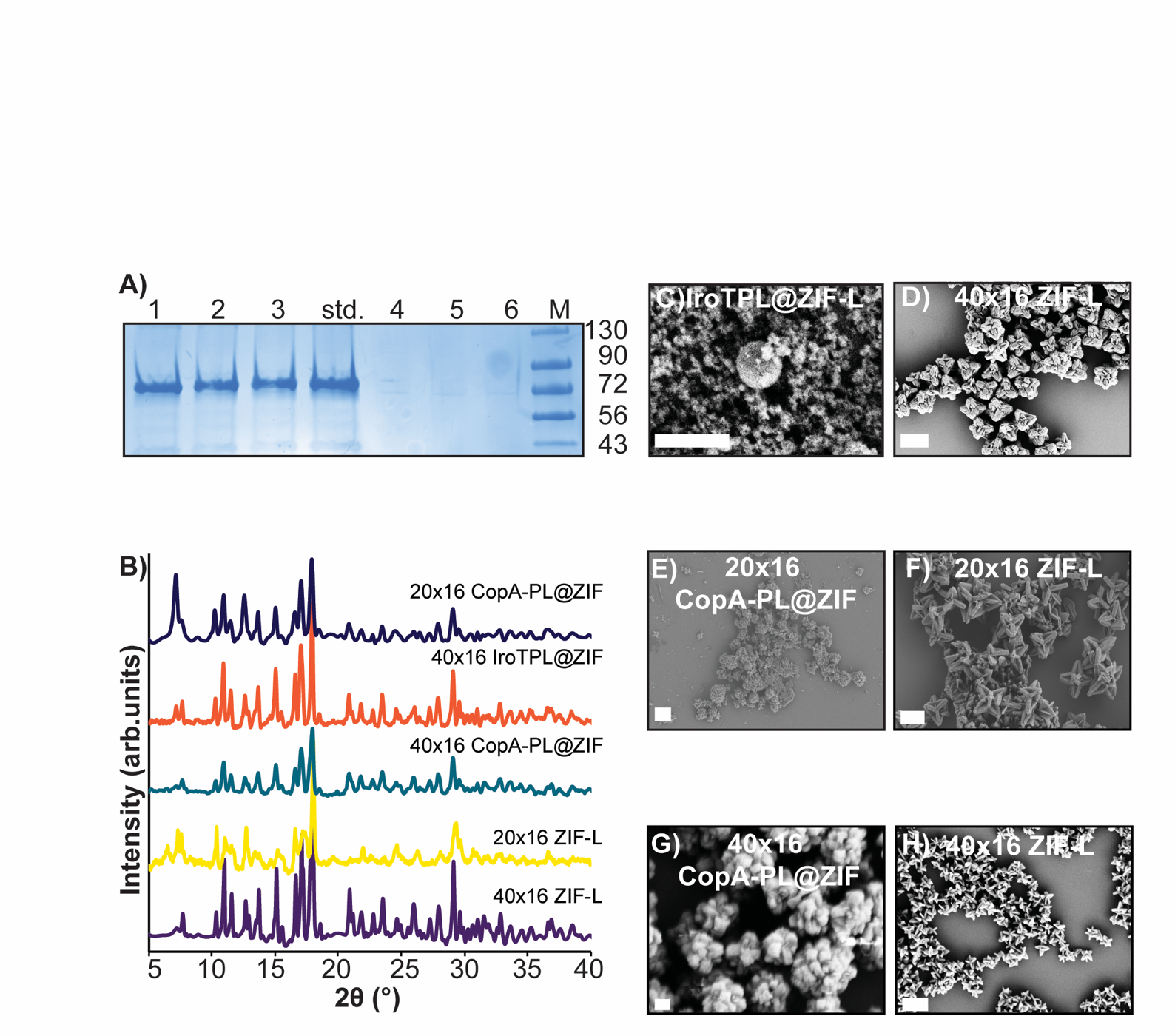


**Supplementary** Figure 16. Characterization of Irot/CopA-PL@ZIF. Encapsulation efficiency quantification via gel densitometry analysis: A) Exfoliated 40*×*16 IroTPL@ZIF. (1) 40*×*16 IroTPL@ZIF at RT 48 h (2) 40*×*16 IroTPL@ZIF at 55 °C (3) 40*×*16 IroTPL@ZIF at 80 °C lanes (4)-(6) supernatants collected for the 40×16 IroTPL@ZIF composites shown in lanes 1-3 B) PXRD spectra of protein-PL@ZIF complexes (CopA-PL@ZIF and IroTPL@ZIF) and ZIF-L controls. Color scheme: 20*×*16 CopA-PL@ZIF (dark-blue), 40*×*16 IroTPL@ZIF (orange), 40*×*16 CopA-PL@ZIF (green), 20×16 ZIF-L (yellow), and 40×16 ZIF-L (purple). ). Y-axis expressed as arbitrary units (arb.units). SEM micrographs of C) 40*×*16 IroTPL@ZIF (Scale bar = 1 µm), D) pristine 40×16 ZIF (Scale bar = 2 µm), E) 20*×*16 CopA-PL@ZIF (Scale bar = 2 µm), F) pristine 20*×*16 ZIF-L (Scale bar = 2 µm), G) 40*×*16 CopA-PL@ZIF (Scale bar = 200 nm), and H) pristine 40*×*16 ZIF (Scale bar = 1 µm).


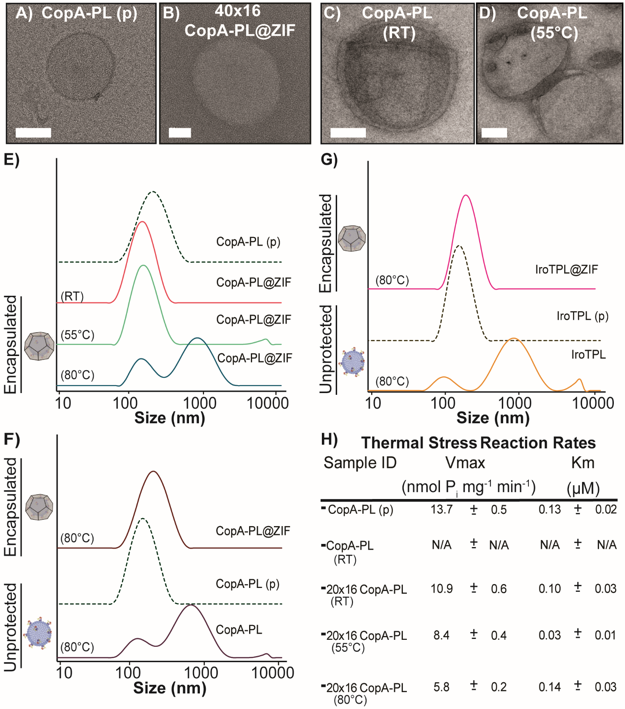


**Supplementary** Figure 17. Characterization of stressed protein-PL@ZIF complexes. TEM micrograph of A) freshly extruded CopA-PL (Scale bar = 100 nm), B) recovered CopA-PL stressed at RT after exfoliation from ZIF composite (Scale bar = 50 nm), C) non-encapsulated CopA-PL stressed at RT (Scale bar = 100 nm), and D) non-encapsulated CopA-PL stressed at 55 ^ͦ^ C (Scale bar =200 nm). DLS of thermally E) stressed 40*×*16 CopA-PL@ZIF. Color scheme: CopA-PL pristine (green), CopA-PL@ZIF RT (red), CopA-PL@ZIF 55 ^ͦ^ C (green), CopA-PL@ZIF 80 ^ͦ^ C (blue). F) DLS of thermally stressed 20*×*16 CopA-PL@ZIF at 80 ^ͦ^ C. Color scheme: CopA-PL pristine (green-dashed line), CopA-PL@ZIF (brown), and unencapsulated control (purple). G) DLS of thermally stressed 40*×*16 IroTPL@ZIF at 80 ^ͦ^ C. Color scheme: IroTPL pristine (green-dashed line), IroTPL@ZIF (magenta), and unencapsulated control (light-orange). H) Thermal stress reaction kinetics recorded for pristine, non-encapsulated, and exfoliated CopA proteoliposomes.


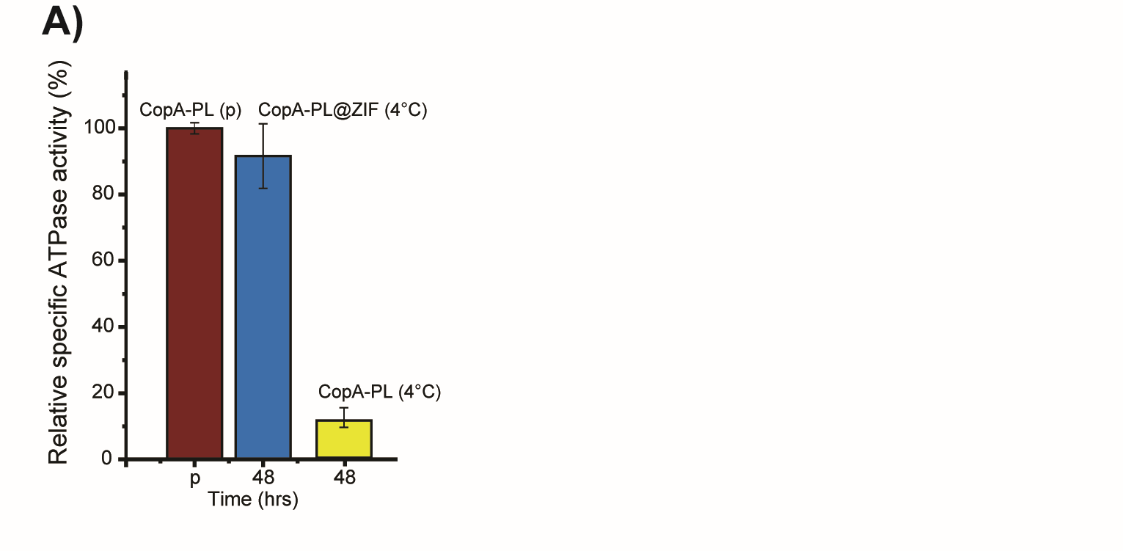


**Supplementary** Figure 18: CopA proteoliposome activity assessment. A) ATPase activity of exfoliated CopA-PL@ZIF (blue) after storage at 4˚C for 48 h. Error bars=standard deviation (n=3). Activity was directly compared against freshly extruded (red) and non-encapsulated CopA-PL (yellow) stored at 4˚C for 48 h.


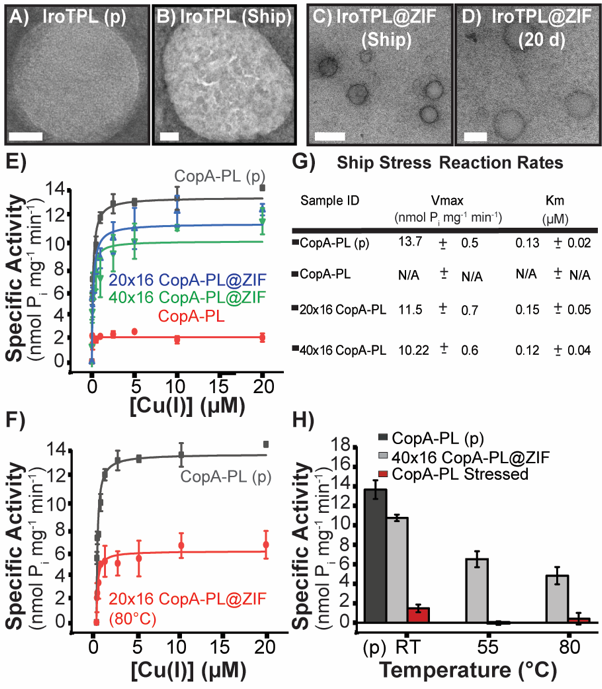


**Supplementary** Figure 19. Specific activity characterization of CopA-PL@ZIF-8 and IroTPL@ZIF-8 bio-composites. TEM micrographs of A) freshly extruded IroTPL (Scale bar =50 nm), B) non-encapsulated IroTPL shipped to Rhode Island from Texas in a USPS bubble cushioned envelope in a small vial containing M-Buffer (Scale bar =200 nm), C) of exfoliated 40*×*16 IroTPL@ZIF shipped to Rhode Island (Scale bar =200 nm), and D) of exfoliated 40*×*16 IroTPL@ZIF stressed at RT for 20 days (Scale bar =100 nm). Activity characterization of E) shipped CopA proteoliposomes. Error bars=standard deviation (n=3). Color scheme: CopA-PL pristine (Gray), exfoliated 40*×*16 CopA-PL@ZIF (green), 20*×*16 CopA-PL@ZIF (blue), and unencapsulated control (red line). F) Activity of exfoliated 20*×*16 CopA-PL@ZIF (red) stressed at 80 °C compared to freshly extruded CopA-PL (grey). Error bars=standard deviation (n=3). G) Calculated ATPase rates for shipped exfoliated CopA proteoliposomes. Rates are directly compared to pristine non-shipped and non-encapsulated shipped CopA proteoliposomes. H) Specific activity of exfoliated 40*×*16 CopA-PL@ZIF (light-grey) thermally stressed bio-composites. Error bars=standard deviation (n=3). Controls include freshly extruded CopA-PL (dark-grey) and unencapsulated control (red).

#
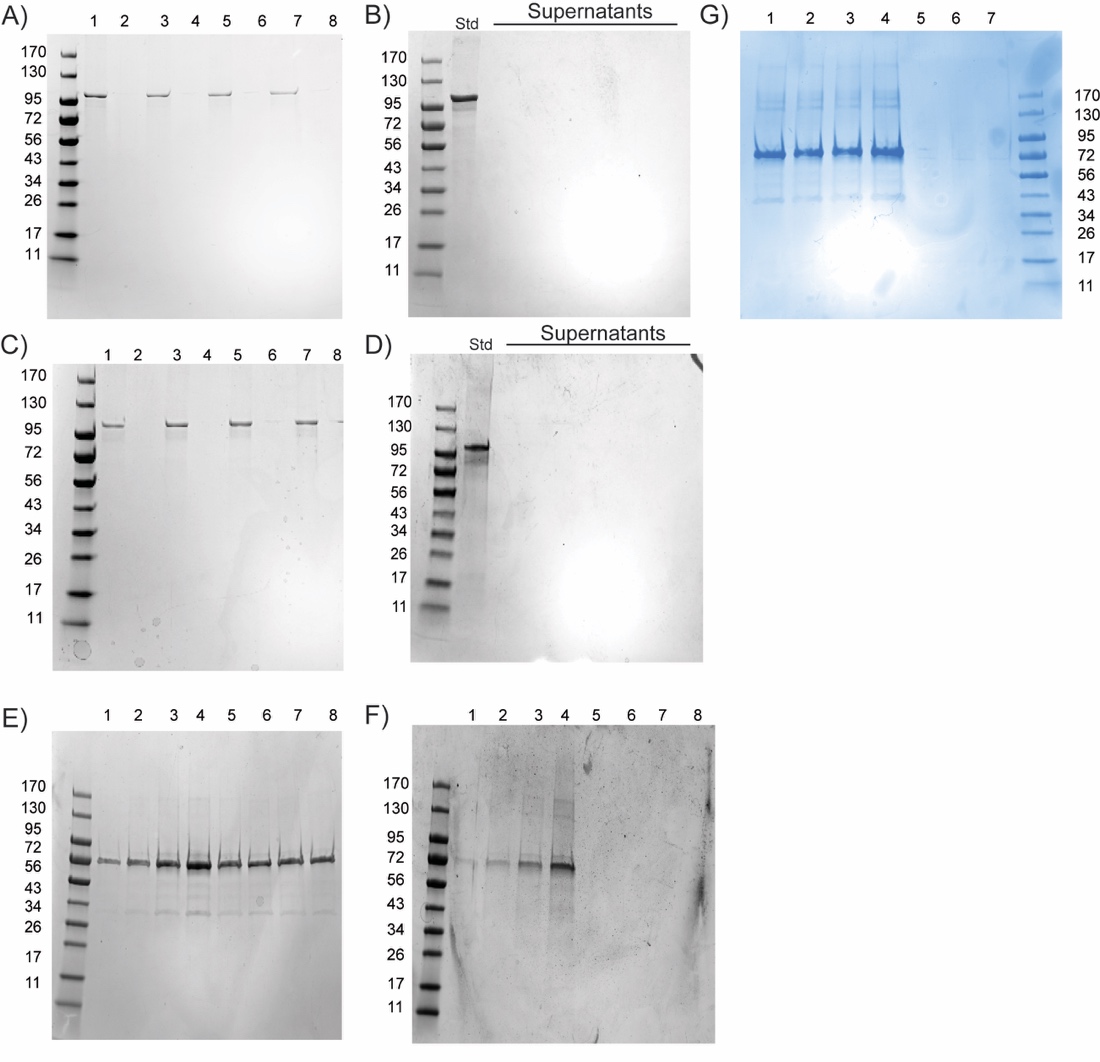


**Supplementary** Figure 20. Full SDS gel images used for determination of protein and proteoliposomes encapsulation efficiency. A) SDS gel of exfoliated: 20×16 CopA@ZIF (1) 20×16 CopA@ZIF stressed at RT (2) 20×16 ZIF-L control RT(3), 20×16 CopA@ZIF stressed at 55 °C (4) 20×16 ZIF-L control stressed at 55 °C (5) 20×16 CopA@ZIF stressed at 80 °C (6), 20×16 ZIF-L control stressed at 80 °C (7) 20×16 CopA@ZIF dried (8) 20×16 ZIF-L control dried. B) SDS gel of exfoliated: 40×16 CopA@ZIF (1) 40×16 CopA@ZIF stressed at RT (2) 40×16 ZIF-L control RT (3), 40×16 CopA@ZIF stressed at 55 °C (4) 40×16 ZIF-L control stressed at 55 °C (5) 40×16 CopA@ZIF stressed at 80 °C (6) 40×16 ZIF-L control stressed at 80 °C (7) 40×16 CopA@ZIF dried (8) 40×16 ZIF-L control dried. C) SDS-PAGE gel of (1) CopA pristine and (2)-(8)supernatants collected for the 20×16 CopA@ZIF composites shown in gel A. D) SDS gel of (1)CopA pristine and (2)-(8) supernatants collected for the 40×16 CopA@ZIF composites shown in gel B. E) SDS-PAGE gel of IroT pristine and exfoliated 40*×*16 IroT@ZIF. (1) 0.062 mg mL^-1^ IroT pristine (2) 0.125 mg mL^-1^ IroT pristine (3) 0.25 mg mL^-1^ IroT pristine (4) 0.5 mg mL^-1^ IroT pristine (5) 40×16 IroT@ZIF at RT 48 h, (6) 40×16 IroT@ZIF stressed at 55 °C (7) 40×16 IroT@ZIF stressed at 55 °C, (8). Non-encapsulated pristine IroT at RT 48 h. F) SDS gel of IroT pristine and supernatants of 40×16 IroT@ZIF bio-composites (1) 0.062 mg mL^-1^ IroT pristine, (2) 0.125 mg mL^-1^ IroTPL pristine, (3) 0.25 mg mL^-1^ IroT pristine, (4) 0.5 mg mL^-1^ IroTpristine (5) 40×16 IroT@ZIF dried supernatant, (6) 40×16 IroT@ZIF at RT 48 h supernatant, (7) 40×16 IroT@ZIF stressed at 55 °C supernatant, (8) 40×16 IroT@ZIF stressed at 80 °C supernatant. G) SDS gel of freshly extruded IroTPL, exfoliated 40×16 IroTPL@ZIF and supernatants of 40×16 IroTPL@ZIF encapsulation. (1) 40*×*16 IroTPL@ZIF at RT 48 h (2) 40*×*16 IroTPL@ZIF at 55 °C (3) 40*×*16 IroTPL@ZIF at 80 °C, (4) freshly extruded IroTPL 0.5 mg mL^-1^, (5) 40×16 IroTPL@ZIF at RT 48 h supernatant, (6) 40×16 IroTPL@ZIF stressed at 55 °C supernatant, (7) 40×16 IroTPL@ZIF stressed at 80 °C supernatant. G) Exfoliated 40*×*16 IroTPL@ZIF (**1**) 40*×*16 IroTPL@ZIF at RT 48 h (**2**) 40*×*16 IroTPL@ZIF at 55 °C (**3**) 40*×*16 IroTPL@ZIF at 80 °C lanes (**4**)-(**6**) supernatants collected for the 40×16 IroTPL@ZIF composites shown in lanes 1-3.

## Supplementary Table 7. Oligos (from 5' to 3') utilized to synthesize the MavN/IroT gene.

| 1 (forward; length: 95 bp)  GTGCACTTGAAGTCCTCTTTCAGGGACCCGGGATGCTGCTGATCATTATCTTCATCTGGTACAAGGTGCTGCACTATGCGAGCATGAGCAAGTAC  2 (reverse; length: 90 bp)  GTCAGCAGCAGCAGGAACAGGTAGTACGGAATTTTCTGCGGCTTAATCGGGATGTTTTTGATGTTCATGGTGGTGTACTTGCTCATGCTC  3 (forward; length: 90 bp)  TCCTGCTGCTGCTGACCGTTGGTGCGAGCCTGATCCTGGGCTTCCTGAGCTTTGGTGGCATGTATGCGCTGTGGCCGGTGCTGCCGCTGG  4 (reverse; length: 90 bp)  GTTCAGCGCACCCTTAATGTTTTGCAGATAGATTTCGCCCTCGTACGCAACGCTCAGCGCAAAACCCGCAAACGCCAGCGGCAGCACCGG  5 (forward; length: 90 bp)  TAAGGGTGCGCTGAACAAGCTGTTCAAATTTAACTACCTGAAGAACTATCTGGCGAAAGAGTACCTGCTGAACCACTTCCCGAACACCGA  6 (reverse; length: 90 bp)  CTTTGTGACCAAACTCCGCCAGCAGGGTCAGTTGTTTCTTATAGTCCTTAAAGAACTGCGGGCAGTTTTCCTCATCGGTGTTCGGGAAGT  7 (forward; length: 90 bp)  GAGTTTGGTCACAAAGAACTGAACCAGGACAGCAAGAAACGTAAACGTCAAATCGAGAAAACCCTGAGCGATATGGAAAAATGGTTCGCG  8 (reverse; length: 90 bp)  AGCCAGTCCAGCAGCTCAATGGTGTATTTGCTGGTGTTCGGGCTCGGGTTGTTCTTGTCGCTAAACAGCTGCAGCGCGAACCATTTTTCC  9 (forward; length: 90 bp)  AGCTGCTGGACTGGCTGGCGCTGAACAAGCGTGATGAGTGGATCAAGCAGTATGAAAAACGTCACTTCCAATTTCACATTGTGAAAGGTT  10 (reverse; length: 90 bp)  CGGGATAATGGTGAACGCTTCAACGATCAGGTAGGTGCTGCCCAGACCCATAAACAGGCCCGCAACCACGCTGAAACCTTTCACAATGTG  11 (forward; length: 90 bp)  GTTCACCATTATCCCGTTCTTTGCGACCATTCCGTTTACCCTGTGGCCGATTATCATTCTGCCGATGGCGACCGTGGCGGGTGCGGCGTA  12 (reverse; length: 90 bp)  GAATTTTGTTGTACCACTTAACCACGGTGTTGTTGTTGATCATATCGGTAACCGCGTTATAGGTCAGCATGCCGTACGCCGCACCCGCCA  13 (forward; length: 90 bp)  TGGTACAACAAAATTCGTAACGACCTGAGCCAGGGTCTGACCGTGCGTAACGTTTTCATCGCGACCACCGCGGTGTTTCTGGTTGGCCTG  14 (reverse; length: 90 bp)  CAATCGAACAGCGGACGCGCGTTGGTCGCAATGGTCCACCAGGTACCCGCGGTGCACACGGTCAGCGCCAGCGCCAGGCCAACCAGAAAC  15 (forward; length: 90 bp)  GTCCGCTGTTCGATTGGATGAAGAAAATGCCGAGCTTTATTATGGGTATCATTAACCCGATCATTACCGGCGCGAGCGCGATCGTTTTCA  16 (reverse; length: 90 bp)  CTTTTGGAAAACGTTCTTGTTGCTACGGGTCGCTTCATCCACCATGTCCAGGCTCTCCGCGGTGTTCTGAATGTTGAAAACGATCGCGCT  17 (forward; length: 90 bp)  GAACGTTTTCCAAAAGATCTACGAGGCGATTAGCAACGGCTATCAGCACCTGCGTGAAACCGAAAACTGGCTGCAAATCGTGAACCCGTT  18 (reverse; length: 90 bp)  CCGCCACGCTGATCAGGTGGCCCAGAAACAGCAGAATACGCAGCGGGGTGATGGTCAGCTTCAGCAGAATACGGAACGGGTTCACGATTT  19 (forward; length: 91 bp)  CTGATCAGCGTGGCGGTTACCAGCGACCGTATGCCGGGTGTGCCGCAGATTCTGAGCGCGCTGGTTGCGATCATTAGCGAGGGCTTCGAAG  20 (reverse; length: 90 bp)  TTGGTGGTGGTCCTGGTGACCGTGTTCCTCATCCAGTTCGTCCTCGTGGGTGTGACCGATAAAGTAGTGCGCATCTTCGAAGCCCTCGCT  21 (forward; length: 90 bp)  CCAGGACCACCACCAATTCGAGAAGCTGCTGAAAGAACGTCTGGACCCGGATAGCGACCAAGATCACAACATGGATATCCCGACCTGGAT  22 (reverse; length: 90 bp)  TCGGGTTCAGCTTGCTCGCGCTGCAGTCCCACAGCGCCGCCAGGCCATAAATCGGGCTCGCGATGGTTTTCAGAATCCAGGTCGGGATAT  23 (forward; length: 90 bp)  AGCAAGCTGAACCCGAGCCAGGAGAACCACAGCAAAAACCTGCCGAGCGGTAAGAAACCGCACGTGCTGAGCCTGGGTGAAGCGTGGAAC  24 (reverse; length: 90 bp)  TGCCACTCCTGGCTCGGACGCTTCGCATGGGTGCTAAATTCAACGTTGAACTCTTTCGCCACGCCCCATTGCTTGTTCCACGCTTCACCC  25 (forward; length: 93 bp)  CGAGCCAGGAGTGGCAAGTGGAACACGCGGTTGCGCAGATCGACAAATTCCAGCGTAAGCACCTGAAAGATATCGTTATTGGCCGTGAGCTGG  26 (reverse; length: 95 bp)  GTATCGCTCAGGGTTTCACCCTTCTCCGGGTGACGGATTTTTTCCTTCAGGTGGTTCAGCGCAATGATTTTCTTGTCCGCCAGCTCACGGCCAAT  27 (forward; length: 95 bp)  AAACCCTGAGCGATACCCTGGAACAGGCGAAGAAACAACCGGACTACAACCAGCACCGTCTGTTCAACCAAAAGGGCGAGAAAACCCGTACCCAG  28 (reverse; length: 95 bp)  CGGAACTACCGCGTGGCACCAGAGCGAGCTCTTTACCGCTGCTCAGGTTAATACGTTCCGGCAGTTCCTCGATAAACAGCTGGGTACGGGTTTTC |
| --- |

##

## Supplementary Table 8. Oligos (from 5' to 3') utilized to synthesize the CopA gene.

| 1A_1 (forward; length: 70 bp)  TCGAAAAGGGTGCACTTGAAGTCCTCTTTCAGGGACCCGGGATGAGCCAAACCATTGATCTGACCCTGGA  1A_2 (reverse; length: 80 bp)  CCACGTCCGGGCGCTGTTCCAGGCTTTCTTTCACACGCTTCACGCAATGGCCGCAGCTCAGACCGTCCAGGGTCAGATCA  1A_3 (forward; length: 80 bp;)  AGCGCCCGGACGTGGAACAGGCGGATGTTAGCATCACCGAGGCGCATGTTACCGGTACCGCGAGCGCGGAGCAGCTGATC  1A_4 (reverse; length: 80 bp)  TCCGCCAGCGGTTTCGCCTTCGGGTGGCTCACGCTCGCATCATAACCCGCTTGCTTAATGGTTTCGATCAGCTGCTCCGC  1A_5 (forward; length: 80 bp)  GAAACCGCTGGCGGAAAGCAGCATTCCGAGCGAGGCGCTGACCGCGGTTAGCGAAGCGCTGCCGGCGGCGACCGCGGATG  1A_6 (reverse; length: 82 bp)  TTCTGAACACGGGTCACGCAGCTCGCGCAGCTCATACCGCTCAGCAGCAGTTGCTGGCTATCATCATCATCCGCGGTCGCCG  1A_7 (forward; length: 100 bp)  GACCCGTGTTCAGAACGCGCTGCAAAGCGTGCCGGGCGTTACCCAGGCGCGTGTGAACCTGGCGGAACGTACCGCGCTGGTTATGGGTAGCGCGAGCCCG  1A_8 (reverse; length: 100 bp)  CTTGCTGACGCTCACGACGTTTCGCGTCGTCTTCGATCGCCTCCGCACCATAGCCCGCCTTCTCAACCGCTTGCACCAGATCCTGCGGGCTCGCGCTACC  1A_9 (forward; length: 100 bp)  GTGAGCGTCAGCAAGAAACCGCGGTGGCGACCATGAAGCGTTTTCGTTGGCAGGCGATTGTGGCGCTGGCGGTTGGTATTCCGGTGATGGTTTGGGGCAT  1A_10 (reverse; length: 100 bp)  GAAAACCATCACCGCCAGGGTAATCAGGCCGATAACCAGCCACAGGCTACGGTTATCCGCGGTCACCATCATGTTGTCACCGATCATGCCCCAAACCATC  1A_11 (forward; length: 100 bp)  GCGGTGATGGTTTTCGCGGGTGGCCACTTTTACCGTAGCGCGTGGAAAAGCCTGCTGAACGGTGCGGCGACCATGGATACCCTGGTGGCGCTGGGTACCG  1A_12 (reverse; length: 100 bp)  CTCGCCTCATAGTACAGGTGACGCGCTTCCATCGGAAACCACTGCGGCCACAGGTTAACGCTCATGCTATACAGCCACGCAACACCGGTACCCAGCGCCA  1A_13 (forward; length: 100 bp)  GTACTATGAGGCGAGCGCGATGATCATTGGCCTGATCAACCTGGGTCACATGCTGGAAGCGCGTGCGCGTCAACGTAGCAGCAAGGCGCTGGAGAAGCTG  1A_14 (reverse; length: 100 bp)  GCATACCCGGCTGAACTTCCGCCAGCGGCACGCTCTTTTCGCCCTCATCGGTCACCAGACGCGCGGTCGGCGGGGTCAGGTCCAGCAGCTTCTCCAGCGC  1A_15 (forward; length: 100 bp)  TTCAGCCGGGTATGCTGCTGCGTCTGACCACCGGTGACCGTGTGCCGGTTGATGGCGAGATCACCCAAGGTGAAGCGTGGCTGGACGAGGCGATGCTGAC  1A_16 (reverse; length: 100 bp)  ACGAAACAGCACGCTACCATCCTGAACCACGGTACCCGCATGCACGCTGTCACCTTCACCTTTTTGCTGCGGAATCGGTTCACCGGTCAGCATCGCCTCG  1A_17 (forward; length: 94 bp)  AGCGTGCTGTTTCGTGCGAGCGCGGTTGGCAGCCACACCACCCTGAGCCGTATCATTCGTATGGTTCGTCAGGCGCAAAGCAGCAAGCCGGAGA  1A_18 (reverse; length: 80 bp)  CACCAGCGCGATAACCACAACCACCGGAACAAACACCGCGCTAATTTTATCCGCCAGCTGACCGATCTCCGGCTTGCTGC  1B_1 (forward; length: 70 bp)  CGCGGTGTTTGTTCCGGTGGTTGTGGTTATCGCGCTGGTGAGCGCGGCGATTTGGTACTTCTTTGGTCCG  1B_2 (reverse; length: 94 bp)  TCGCCAGGCCCAGCGCGCACGGGCACGCAATGATCAGCACGGTGGTCGCAATAACCAGGGTATACACGATTTGCGGCGCCGGACCAAAGAAGTA  1B_3 (forward; length: 80 bp)  CGCTGGGCCTGGCGACCCCGATGAGCATCATTAGCGGCGTTGGTCGTGCGGCGGAATTCGGTGTGCTGGTTCGTGATGCG  1B_4 (reverse; length: 80 bp)  TCGGTCAGGGTGCCGGTCTTATCGAAAACCACGGTGTCCAGGGTGCTCGCACGCTGCAGCGCATCCGCATCACGAACCAG  1B_5 (forward; length: 80 bp)  CGGCACCCTGACCGAGGGCAAGCCGCAAGTGGTTGCGGTGAAAACCTTTGCGGACGTTGATGAAGCGCAAGCGCTGCGTC  1B_6 (reverse; length: 83 bp)  ATCGCCCGCTTTGTCCAGGATCGCACGCGCCAGCGGGTGGCTGCTACCCTGTTCCAGCGCCGCCGCCAGACGCAGCGCTTGCG  1B_7 (forward; length: 100 bp)  GACAAAGCGGGCGATATGCAGCTGCCGCAAGTGAACGGTTTTCGTACCCTGCGTGGCCTGGGTGTTAGCGGTGAAGCGGAAGGTCATGCGCTGCTGCTGG  1B_8 (reverse; length: 100 bp)  GTCGCACCTTGGCTCGCCTGCGCGGTAATTTCCGCCTCGATCGCCTTGGTACCAACTTGCTGTTCGTTCAGCAGCGCCTGGTTACCCAGCAGCAGCGCAT  1B_9 (forward; length: 100 bp)  GAGCCAAGGTGCGACCCCGGTGCTGCTGGCGGTTGACGGTAAAGCGGTGGCGCTGCTGGCGGTTCGTGACCCGCTGCGTAGCGATAGCGTTGCGGCGCTG  1B_10 (reverse; length: 100 bp)  CCGCTTCTTTCGCGATCGCGTTCGCGGTGGTCGGGTTATCACCGGTCAGCATAACCAGACGGTAGCCCGCCTTGTGCAGACGTTGCAGCGCCGCAACGCT  1B_11 (forward; length: 100 bp)  TCGCGAAAGAAGCGGGTATCGACGAAGTGATTGCGGGCGTTCTGCCGGATGGCAAGGCGGAAGCGATTAAACACCTGCAGAGCGAGGGCCGTCAAGTGGC  1B_12 (reverse; length: 100 bp)  CGCAACATCGCTACCGCCACCCATCGCAATACCCACATCCGCCTGCGCCAGCGCCGGCGCATCGTTGATACCGTCGCCAACCATCGCCACTTGACGGCCC  1B_13 (forward; length: 100 bp)  GGTAGCGATGTTGCGATCGAAACCGCGGCGATTACCCTGATGCGTCATAGCCTGATGGGCGTTGCGGATGCGCTGGCGATCAGCCGTGCGACCCTGCACA  1B_14 (reverse; length: 100 bp)  CCGGTAAACGGCCACAGAATACCCGCCGCCACCGGAATGCCGATGCTGTTATAGATGAACGCACCCAGCAGGTTTTGTTTCATGTTGTGCAGGGTCGCAC  1B_15 (forward; length: 94 bp)  GTGGCCGTTTACCGGTACCCTGCTGAACCCGGTGGTTGCGGGTGCGGCGATGGCGCTGAGCAGCATCACCGTTGTTAGCAATGCGAACCGCCTG  1B_16 (reverse; length: 80 bp)  TGGTGATGAGCGGAACTACCGCGTGGCACCAGAGCGAGCTCTTACTCCTTCGGCTTGAAACGCAGCAGGCGGTTCGCATT |
| --- |
